# The Effects of Satisfaction of Basic Psychological Needs at School on Children’s Prosocial Behavior and Antisocial Behavior: The Mediating Role of School Satisfaction

**DOI:** 10.3389/fpsyg.2018.00548

**Published:** 2018-04-17

**Authors:** Lili Tian, Xiao Zhang, E. Scott Huebner

**Affiliations:** ^1^School of Psychology, Guangdong Key Laboratory of Mental Health and Cognitive Science, Center for Studies of Psychological Application, South China Normal University, Guangzhou, China; ^2^Department of Psychology, University of South Carolina, Columbia, SC, United States

**Keywords:** satisfaction of basic psychological needs at school, school satisfaction, prosocial behavior, antisocial behavior, mediation, children

## Abstract

Grounded in Basic Psychological Need Theory, we examined the direct effects of the satisfaction of three basic psychological needs at school (i.e., satisfaction of autonomy needs at school, satisfaction of relatedness needs at school, and satisfaction of competence needs at school) on prosocial behavior and antisocial behavior as well as the mediation effects of school satisfaction on the relations between the satisfaction of three basic psychological needs at school and prosocial behavior as well as antisocial behavior. We employed a sample of 801 Chinese children (429 males; *M*age = 9.47) in a three-wave longitudinal study, with each wave occurring 6 months apart. Direct and indirect effects were estimated by Structural Equation Modeling. Results indicated that: (1) Satisfaction of relatedness needs at school and competence needs at school, but not satisfaction of autonomy needs at school, displayed direct effects on prosocial behavior. Also, satisfaction of relatedness needs at school, but not satisfaction of autonomy needs at school or competence needs at school, displayed direct effects on antisocial behavior. (2) Both satisfaction of relatedness needs at school and competence needs at school displayed indirect effects on prosocial behavior and antisocial behavior via school satisfaction as a mediator. However, satisfaction of autonomy needs at school failed to have indirect effects on prosocial behavior or antisocial behavior via school satisfaction. These findings suggest differential predictors of children’s prosocial and antisocial behavior, supporting the separability of the two constructs. The findings also suggest developmental differences in need satisfaction, with the satisfaction of autonomy needs playing a relatively less important role in school-age children. We also discussed limitations and practical applications of the study.

## Introduction

Children’s social behavior has been a topic of great interest to researchers and educators, including prosocial behavior and antisocial behavior (Crowley and Merrell, 2003; Raimundo et al., 2012). Prosocial behavior as a representative of positive social behavior is critical for children’s social adjustment and physical and mental health (Dong and Lin, 2011). Children’s differences in prosocial behavior partly arise from early interaction experiences and childhood environments (Penner et al., 2005). With increasing age, children generally express more prosocial behavior and/or increased complexity in prosocial responding, probably due to improvements in their cognitive abilities, socio-emotional skills, and physical abilities at the time of entry into elementary school (Zahn-Waxler et al., 1983; Eisenberg and Fabes, 1998). To the contrary, some children show increased antisocial behavior after entry into elementary school. The terms of antisocial behavior and prosocial behavior refer to the moral inhibitive and proactive aspects (Kavussanu and Boardley, 2009). Antisocial behavior hinders appropriate socialization of children, leading to difficulties in controlling their behavior, making friends, and succeeding in school (Odgers et al., 2012; Pears et al., 2016). Developmental models of antisocial behavior conceptualize antisocial behavior as often trait-like, emerging early in life and lasting into adolescence and adulthood (Patterson et al., 1989). If antisocial behavior continues across childhood, it yields costly physical health, mental health, social, and economic difficulties (Moffitt et al., 2002; Cohen and Piquero, 2009).

Basic Psychological Need Theory (BPNT) is a sub-theory of Self-Determination Theory (SDT; Deci and Ryan, 1985, 2000), proposing that human beings are born with three basic psychological needs: the needs for autonomy, competence, and relatedness. These needs are not learned but are an aspect of human nature and thus operate across differences in gender, culture, and time (Deci and Vansteenkiste, 2004), including childhood and adulthood (Véronneau et al., 2005). Contexts, such as school, are thought to support or inhibit the satisfaction of these needs. More specifically, on the one hand, children in supportive environments should be able to more easily satisfy basic psychological needs, increasing the likelihood of experiencing well-being and healthy development, as well as behaving in a prosocial manner (Deci and Ryan, 2000). On the other hand, children in non-supportive environments should be less able to satisfy their needs, increasing the likelihood of maladaptive behavior (Shields et al., 2001; Deci and Ryan, 2011), as well as behaving in an antisocial manner (Thibodeau et al., 2015).

Children spend most of their daytime in school. Thus, schools likely influence both children’s basic psychological needs and their behaviors related to need satisfaction, both positive and negative. Schools also set the standards for the patterns of behaviors and attitudes of school-age children. Several reviews of studies about school contextual effects on students’ outcomes suggest that the psychosocial component of the school environment may be the most important component (Sellström and Bremberg, 2006; Fletcher et al., 2008). Understanding how children’s satisfaction of basic psychological needs at school affects their prosocial and antisocial behavior should not only contribute to the BPNT, but it should also inform empirically-validated approaches for eliciting children’s prosocial behavior and intervening to ameliorate or prevent antisocial behavior.

Accordingly, we targeted school-age children for our study, examining whether as well as to what extent children’s basic psychological needs satisfaction at school predict their social behavior (i.e., prosocial behavior and antisocial behavior). We also sought to identify mediating mechanisms that account for the associations.

### Satisfaction of Basic Psychological Needs at School

Human beings display inherently basic psychological needs; namely, the needs for autonomy, relatedness as well as competence, and the satisfaction of these needs plays an important role in general and in specific domains, whether school, work, or any other relevant domain (Deci and Ryan, 1985, 2000; Milyavskaya and Koestner, 2011). Based on the BPNT, Tian et al. (2014b) applied three basic psychological needs to the study of students’ school lives, proposing the construct of *adolescent students’ basic psychological needs at school* to come up with students’ needs satisfaction as experienced specifically during daily school life. In the school context, needs for autonomy represents individual desires to experience the sense of freewill and self-endorsement of their behaviors at school; needs for relatedness represents individual desires to experience the feeling of school belonging, which includes connection with their teachers and peers; needs for competence represents individual desires to interact with the school context effectively and to experience chances for developing and demonstrating individual capabilities. Accordingly, the Adolescent Students’ Basic Psychological Needs at School Scale (ASBPNSS) was developed by Tian et al. (2014b) to measure adolescents’ satisfaction of basic psychological needs at school.

Satisfaction of basic psychological needs is a basic driving force for individual behavior that predicts both performance and well-being outcomes at the individual personality sub-level (i.e., individual differences, motivations, and self) as well as other higher levels (i.e., culture and social relations) (Deci and Ryan, 2000, 2011). Research on the effects of basic psychological needs at school has focused predominantly on predicting subjective well-being and educational performance outcomes (Tian et al., 2014a, 2015), with little attention given to behavioral outcomes in the school domain. Some research has paid attention to the effects of basic psychological needs on individuals’ behavior, but these studies have concentrated almost exclusively on the negative aspects of behavior. For example, children who fail to experience basic psychological needs satisfaction are more likely to display emotional and behavioral dysregulation during peer interactions (Shields et al., 2001). For another example, adolescents who do not experience satisfaction of basic psychological needs are more likely to adopt passive coping styles in their efforts to regulate their smoking and drinking behavior (Xia and Ye, 2014). Relatively few studies have considered basic psychological needs satisfaction in explaining variation in positive behaviors, such as prosocial behavior. For one example, Gagneì (2003) and Haivas et al. (2013) showed that basic needs satisfaction positively predicted prosocial behavior in cross-sectional studies. However, it should be noted that their sample was limited to adolescents and adults, excluding younger children who display rapidly growing needs for autonomy, relatedness, and competence. Because schools can support or thwart need satisfaction, assessments of basic needs satisfaction at school should afford an indication of the quality of the interactions between the children and their school environments, enabling the development of appropriate school-based interventions at an early age (Ryan and Deci, 2004). Thus, we explored the relations between children’s psychological needs satisfaction in school and the development of both prosocial and antisocial behavioral outcomes by using a three-wave longitudinal design, in order to explore psychosocial functioning more comprehensively than in previous studies.

### Prosocial Behavior and Its Relation With Satisfaction of Basic Psychological Needs at School

Prosocial behavior, which was defined as an action aimed to help or benefit others (Eisenberg and Fabes, 1998), includes various specific behaviors; for example sharing, helping, co-operating, volunteering, and donating (Carlo, 2006). These behaviors each have distinctive characteristics, but they all involve intentional actions that help or benefit others, which is why prosocial behavior is often associated with altruism (Schroeder et al., 1995; Batson, 1998; Eisenberg and Fabes, 1998). Many researchers have investigated various underlying motives for these positive behaviors (Finkelstein et al., 2005; Gebauer et al., 2008; Grant, 2008). The motives can be divided into extrinsic motivation and intrinsic motivation. In some cases, researchers have attempted to increase prosocial behavior through the use of external factors, although without much success (Sobus, 1995; Stukas et al., 1999). In other cases, researchers have suggested that internal motives prompting people to help others can influence their experience of and outcomes associated with helping. For example, autonomous motivation and autonomy orientation representing a tendency toward volitional or autonomous engagement in action were proved to be strong predictors of adolescents’ engagement in prosocial activities. Furthermore, the satisfaction of basic psychological needs mediated the relations (Gagneì, 2003; Weinstein and Ryan, 2010). Also, previous findings demonstrated that the satisfaction of relatedness needs increased prosocial motivation and behavior (Pavey et al., 2011). Recent research demonstrated that autonomy need satisfaction and competence need satisfaction positively predicted volunteer’s engagement in work and negatively predicted their intention to quit volunteer work (Haivas et al., 2013). More specifically, Gagneì (2003) found that competence needs were more strongly related to prosocial behavior than autonomy or relatedness needs. Together, these results all support the BPNT, but the directionality of influences between the three basic psychological needs satisfaction and prosocial behavior cannot be inferred from the cross-sectional nature of the studies. Additionally, when children start school and begin to expand their social interaction and social skills, they usually gain more opportunities to participate in prosocial behavior. In the current study, we thus attempted to develop further the research investigating the motives underlying prosocial behavior by considering whether prosocial behavior is a direct outcome of the three basic needs satisfaction in the school setting, using a longitudinal research design.

### Antisocial Behavior and Its Relation With Satisfaction of Basic Psychological Needs at School

In our study, antisocial behavior was defined as consisting of aggressive and delinquent behaviors that result in physical or psychological harm to others or their property (e.g., ‘bullying,’ ‘breaking the rules,’ and ‘getting into fights’). Previous research exploring the antecedents of children’s antisocial behavior has focused on dispositional and socialization factors, such as temperament, character, and social support (Odgers et al., 2012; Brajša-Žganec and Hanzec, 2014; Kerekes et al., 2017). In the past few decades, however, increasing numbers of studies have emphasized the importance of analyzing the individual contributions of these factors combined with the complex interactions of characteristics of the child and social environment that may lead to children’s behavior problems.

Antisocial behavior is connected with social and educational impairments, which can persist in intensity, form, and frequency across development in childhood (Moffitt, 1993; Kim et al., 2014). During the school-age years, children’s social relationships in schools primarily involve their teachers and peers. Impaired social relations with significant others (i.e., teachers and peers) may not only fail to satisfy needs for relatedness, they may, in turn, lead to subsequent antisocial behavior (Gagneì, 2003; Tian et al., 2015). This notion is consistent with the cascade model of social and emotional development (Dodge et al., 2008), which suggests that unsuccessful relationships with teachers and peers in early school years escalate into more serious forms of antisocial behavior in later years (Snyder et al., 2005). Recent studies have also demonstrated that lacking autonomy support, maltreated children show more antisocial behavior (Shields et al., 2001; Thibodeau et al., 2015). Moreover, a coexistence is often observed between antisocial behavior and academic difficulties, compounding children’s difficulties (Pagani et al., 2008, 2017; Wang and Fredricks, 2014). Because increasing social skill deficits and higher frequencies of antisocial behavior have been reported among children (Moffitt et al., 2002; Cohen and Piquero, 2009), increased research and professional practice attention to social behavior in school settings seems necessary. Very few studies have analyzed the effects of individual differences in basic psychological needs satisfaction *at school* on children’s antisocial behavior. Thus, we aimed to extend the extant literature by exploring further the precise linkages between specific satisfaction of psychological needs in school and later children’s antisocial and prosocial behavior simultaneously.

### The Mediating Role of School Satisfaction

As one domain of general life satisfaction, school satisfaction refers to a student’s subjective, cognitive evaluation of the quality of overall school life using her or his internal standards related to several specific school life domains (e.g., achievement, school management, teaching) (Tian et al., 2015). The measure of school satisfaction reflects the degree to which a child is adjusted to the school environment as well as whether the school is providing an environment that promotes psychological health (Baker et al., 2003). Both theoretical and empirical rationales exist for the contention that school satisfaction is influenced by basic psychological needs satisfaction at school. Theoretically, the BPNT posits that fluctuations in need satisfaction will directly predict fluctuations in well-being. As one aspect of *global* subjective well-being (Shin and Johnson, 1978; Diener and Diener, 1995; Veenhoven, 1996), life satisfaction has also been shown to predict by basic psychological needs satisfaction in adolescents and adults. Tian et al. (2014a) further showed that basic psychological needs *at school* also predicted school well-being, including school satisfaction as the cognitive dimension and positive and negative affect as the emotional dimension of school well-being. Furthermore, researchers suggested that students’ evaluations of their school experiences are based on how well schools satisfied basic psychological needs (Connell and Wellborn, 1991). Thus, school satisfaction, as a subjective experience, may provide a personal reflection of how well schools are satisfying the needs for autonomy, relatedness, and competence. For instance, children who are accepted by their peers are more likely to report enjoying school (Osterman, 2000). Additionally, as suggested by Verkuyten and Thijs (2002), the relation between school grades and school satisfaction may be mediated by students’ perceptions of competence, indicating that school satisfaction may be a reflection of the need for perceived competence. Although no studies have been reported on the relation between autonomy and school satisfaction, the life satisfaction literature may provide support for the relation in that adolescents’ life satisfaction was significantly related to their parents’ perceived promotion of autonomy (Suldo and Huebner, 2004).

Although researchers often study school satisfaction as an outcome variable, the literature of life satisfaction provides some support for examining subjective satisfaction experiences as an intervening variable (McKnight et al., 2002; Španić et al., 2011). For example, Španić et al. (2011) discovered that life satisfaction may operate as a cognitive variable that helps account for the relationship between stressful experiences and particular behavior response. Also, life satisfaction has been shown to be closely related to behavioral outcomes in children and adolescents, such as prosocial behavior (Caprara and Steca, 2005; Martin and Huebner, 2007), aggressive behavior (Valois et al., 2001) and problem behavior (MacDonald et al., 2005), and it could further independently influence prosocial behavior in students (Afolabi et al., 2014). Recently, researchers have observed that low school satisfaction contributed to alienation from school and rebellion against school (Takakura et al., 2010) as well as antisocial and other problem behaviors (Haranin et al., 2007; Loukas and Murphy, 2007; Takakura et al., 2010). Thus, children who have higher school satisfaction may be more likely to engage in prosocial behavior whereas children who have lower school satisfaction may be more likely to engage in antisocial behavior. However, relevant research is scanty. Thus, we sought to explore further whether school satisfaction mediates the longitudinal relation between the basic psychological needs satisfaction at school and later children’s prosocial and antisocial behavior.

### The Current Study

The main goal of our study was to explore the longitudinal interrelations among the satisfaction of basic psychological needs at school (i.e., satisfaction of autonomy needs at school, satisfaction of relatedness needs at school, satisfaction of competence needs at school), cognition evaluation factor of subjective well-being (i.e., school satisfaction), and social behavior (i.e., prosocial behavior, antisocial behavior) in elementary school children. Specifically, three hypotheses were formulated: (1) the satisfaction of the three basic psychological needs at school will show positive direct effects on children’s prosocial behavior and negative direct effects on children’s antisocial behavior, (2) children’s school satisfaction will mediate the relation between the satisfaction of the three basic psychological needs at school and children’s prosocial behavior, (3) children’s school satisfaction will mediate the relation between the satisfaction of the three basic psychological needs at school and children’s antisocial behavior. Using three waves of data 6 months apart, we assessed basic psychological needs satisfaction at school at Time 1 (T1), school satisfaction at Time 2 (T2), and prosocial behavior and antisocial behavior at Time 3 (T3).

## Materials and Methods

### Participants

We randomly drew the participants from public elementary schools in a southern city in China. The participants were surveyed on three occasions, 6 months apart. At the baseline assessment (Time 1), 842 children (46.4% females) from Grades 3 to 5 participated, participants’ ages were between 7 and 12 years (*M* = 9.46 years, *SD* = 0.96). At the Time 2 assessment 6 months later, 819 participants (46.5% females) were recruited from the same grades as at Time 1. At the Time 3 assessment, 6 months after Time 2, 801 participants (46.4% females) recruited from Grades 4 to 6 (the original sample was in Grades 4–6 at Time 3) participated and they were retained from the original Time 1 sample. Children who participated in all waves of the project were not significantly different from the participants who had missing data in at least one wave. We did not find differences between the two groups in relation to satisfaction of autonomy needs at school [*t*(840) = 1.61, *p* > 0.05], satisfaction of relatedness needs at school [*t*(840) = 0.49, *p* > 0.05], satisfaction of competence needs at school [*t*(840) = 1.82, *p* > 0.05], or school satisfaction [*t*(817) = -0.42, *p* > 0.05] for the current samples.

The sample was a convenience sample. Nevertheless, based on the information from the local education authorities, the participating schools were all coeducational and comparable in aspects of school size, class size, as well as teachers’ teaching ability. They were all representative of the schools in the region. The majority of the participants came from middle-income families, and their parents had all earned a high school degree or higher.

### Measures

#### Satisfaction of Basic Psychological Needs at School

The ASBPNSS (Tian et al., 2014b) was used to measure the satisfaction of basic psychological needs at school at Time 1. It is a self-report scale with 15 items, which was comprised of three subscales: (a) The need for Autonomy consisting of five items (e.g., ‘I feel like I can pretty much be myself at school.’), (b) The need for Relatedness consisting of five items (e.g., ‘I have few close friends at school.’), and (c) The need for Competence consisting of five items (e.g., ‘Most days I feel a sense of accomplishment from studying at school.’). Participants responded to items by providing a rating ranging from 1 (*strongly disagree*) to 6 (*strongly agree*).

To explore the factor structure of the ASBPNSS in the current study, exploratory factor analyses (EFAs) were performed. The test of Bartlett’s was statistically significant, χ^2^ = 2663.04, *df =* 105, *p* < 0.001. The Kaiser-Meyer-Olkin statistic was 0.88, which is higher than its threshold value. Results showed that the all 15 items loaded satisfactorily on their respective factors (i.e., needs for autonomy; needs for relatedness; needs for competence), accounting for 22.77, 19.54, and 18.76% of the variance respectively for all the participants in this sample. Factor loadings ranged from 0.50 to 0.85.

To determine further the factor structure of the ASBPNSS in the current sample, confirmatory factor analysis (CFA) was performed. The results revealed support for the three-factor structure for basic psychological needs satisfaction at school, χ^2^(86) = 448.94; CFI = 0.93; TLI = 0.92; SRMR = 0.05; RMSEA = 0.07. For the subscales of autonomy needs, relatedness needs, and competence needs, the Cronbach’s alpha coefficients were respectively 0.87, 0.83, and 0.80 for the sample at T1. According to all these indexes, the reliability of the ASBPNSS is acceptable for school-age children in the current study.

#### Prosocial Behavior and Antisocial Behavior

##### Prosocial behavior

Prosocial behavior was measured by the Altruistic Behavior subscale from the Primary School Upper Grade Students’ Prosocial Behaviors Questionnaire (PSUGSPBQ; Li, 2009) at Time 3, which consists of eight items (e.g., ‘If my classmates have difficulties in study which I am able to work out, I will teach them.’). Participants responded to items by providing a frequency rating ranging from 1 (*never*) to 5 (*always*). Higher scores indicated more frequent prosocial behavior. The Altruistic Behavior subscale shows sound psychometric properties with Chinese elementary school students (Tian et al., 2016). A CFA was performed to determine the factor structure of the Altruistic Behavior subscale in the current sample. The results supported its expected one-factor structure for, χ^2^(12) = 40.04; CFI = 0.99; TLI = 0.98; SRMR = 0.02; RMSEA = 0.05. This subscale displayed high internal consistency (coefficient α = 0.90) at Time 3.

##### Antisocial behavior

Antisocial behavior was measured at Time 3 by the Antisocial Behavior subscale from the Left-behind Children’s Social Behavior Questionnaire (LCSBQ; Chen, 2008), which consists of five items (e.g., ‘Violating discipline or regulation in school’). Participants responded to items by providing a frequency rating ranging from 1 (*never*) to 4 (*often*). Lower scores indicated more frequent actions that are not considered acceptable and approved by society (i.e., antisocial behavior). The Antisocial Behavior subscale shows sound psychometric properties with Chinese elementary school students (Chen, 2008). A CFA was performed to determine the factor structure of antisocial behavior in the current sample. The results provided support for the expected one-factor structure, χ^2^(4) = 20.30; CFI = 0.99; TLI = 0.98; SRMR = 0.02; RMSEA = 0.07. This subscale demonstrated high internal consistency (coefficient α = 0.81).

#### School Satisfaction

The School Satisfaction subscale of the Brief Adolescents’ Subjective Well-Being in School Scale (BASWBSS; Tian et al., 2015) was used at Time 2 to measure school satisfaction. It consists of six items (e.g., ‘The curriculum and homework assigned are reasonable.’). This subscale covers six dimensions of students’ school life: academic learning, school management, teacher–student relationships, achievement, peer relationships and teaching. Participants responded to items by providing a rating ranging from 1 (*strongly disagree*) to 6 (*strongly agree*). Higher scores indicated higher levels of school satisfaction. The School Satisfaction subscale has shown sound psychometric properties with Chinese elementary school students (Tian et al., 2016). A CFA was performed to determine the factor structure of the School Satisfaction subscale with the current sample. The results provided support for its expected one-factor structure, χ^2^(6) = 26.91; CFI = 0.99; TLI = 0.97; SRMR = 0.02; RMSEA = 0.07. This subscale had high internal consistency (coefficient α = 0.85).

### Procedure

Consistent with institutional review board procedures in China, parental consent and participant assent were obtained before participation. According to the procedures approved by the Human Research Ethics Committee of South China Normal University, all students were considered voluntary for the three waves of data collection. Only students who provided their own assent and their parents’ consent forms were allowed to participate in our study, and they had the right to drop out at any time during the administration of the survey measures. The surveys were administered in the same manner to groups of about 45 students by two trained graduate assistants at each time point, 6 months apart. Identical verbal and written instructions and time requirements for completing the measures were given to the participants. After finishing all the measures, participants were debriefed regarding the purpose of the investigation. Additionally, participants were asked to provide sociodemographic information, such as age, grade, and gender.

### Data Analyses

Taking into account the relatively large sample size and small amount of missing data (i.e., 4.87%), we handled the missing data by using the list-wise deletion procedure. This is an acceptable method when the missing data is less than 5% of the total lost cases (Graham, 2009). The data were analyzed via the following five steps. First, Harman’s one-factor test (Podsakoff and Organ, 1986) was run to test for common method variance (CMV). Second, descriptive statistics and correlational analyses were performed by using the SPSS 16.0 statistical package (Norusis, 2008). Third, structural equation modeling (SEM) in Mplus 7.0 statistical package (Muthén and Muthén, 2012) was run to examine a model in which T1 satisfaction of basic psychological needs at school were all expected to be direct predictors of T3 prosocial and antisocial behavior. Fourth, SEM in Mplus 7.0 was also used to analyze the mediation effects of school satisfaction. To examine the mediation effects and bias-corrected percentile confidence intervals (CIs), the bootstrapping test was used, employing 1000 samples (MacKinnon, 2008).

We used multiple indicators to analyze SEM fit. At first, χ^2^ was considered. Conventionally, the non-significant χ^2^ likelihood ratio means a good factor structure. However, other goodness-of-fit-measures were also considered because of the sensitivity of the χ^2^ statistic to sample size (Saris, 1982; Jöreskog and Sörbom, 1996). These measures included the Tucker-Lewis index (TLI), the comparative fit index (CFI), standardized root mean square residual (SRMR), and the root mean square error of approximation (RMSEA). With regard to the CFI and TLI indices, values greater than 0.95 indicated good fitting models, and values greater than 0.90 indicated acceptable and adequate fitting models (Hu and Bentler, 1999; Byrne, 2013). For the SRMR, values less than 0.06 were considered indicative of good fit, while values less than 0.08 were considered indicative of adequate fit (Hu and Bentler, 1999; Marsh et al., 2004). As to the RMSEA, values less than 0.06 were considered indicative of good fit, while values between 0.06 and 0.10 were considered adequate fit (Hu and Bentler, 1999; Kaplan, 2000).

## Results

### Assessment of Common Method Variance

Before testing the hypotheses, we addressed the issue of CMV by conducting a Harman’s one-factor test (Podsakoff and Organ, 1986), which used EFA to determine the extent of CMV in data. All of the variables of interest were entered into an EFA in the procedure. If a substantial amount of CMV is present, either a single factor will emerge from the factor analysis, or one general factor will account for most of the variance (>40%). In the current study, the EFA resulted in seven factors with eigenvalues greater than 1; the first factor accounted for only 26.64% of total variance, indicating no significant CMV in our data. Although we were unable to rule out fully any contamination problems caused by CMV, the results of these relatively stringent analyses help strengthen confidence in our findings.

### Descriptive Statistics and Correlations

Descriptive statistics and correlations among study variables are summarized in **Table 1**. Univariate skewness was less than 2, and univariate kurtosis was less than 7.0, which indicated no non-normality in the data (Muthén and Kaplan, 1992; Curran et al., 1996). Each of the T1 basic psychological needs satisfaction were positively related to T3 prosocial behavior and T2 school satisfaction respectively. T1 relatedness needs satisfaction at school and competence needs satisfaction at school were negatively related to T3 antisocial behavior, whereas T1 autonomy needs satisfaction at school was not significantly related to T3 antisocial behavior. T2 school satisfaction was positively related to T3 prosocial behavior and negatively related to T3 antisocial behavior.

**Table 1:** Descriptive statistics and correlations for the observed variables (*N* = 801).

Variable	*M*	*SD*	Skewness	Kurtosis	1	2	3	4	5	6	7
(1) T1 Auto	4.00	1.42	-0.36	-0.91	_						
(2) T1 Rela	5.37	0.87	-1.79	3.21	0.27^∗∗^	_					
(3) T1 Comp	5.06	0.89	-1.43	2.05	0.31^∗∗^	0.58^∗∗^	_				
(4) T2 SS	5.27	0.82	-1.63	3.03	0.17^∗∗^	0.48^∗∗^	0.45^∗∗^	_			
(5) T3 Prosocial Behavior	33.84	6.28	-0.94	0.39	0.12^∗∗^	0.38^∗∗^	0.37^∗∗^	-0.39^∗∗^	_		
(6) T3 Antisocial Behavior	6.62	2.14	1.90	4.54	-0.05	-0.26^∗∗^	-0.21^∗∗^	-0.37^∗∗^	-0.34^∗∗^	_	
(7) Gender	1.47	0.50	0.14	-2.00	0.05	0.06	0.02	0.01	0.02	-0.25^∗∗^	_
(8) Age	9.46	0.96	-0.01	-0.90	-0.02	-0.07	-0.03	-0.15^∗∗^	-0.04	-0.11^∗∗^	-0.07

*Auto = satisfaction of autonomy needs at school; Rela *=* satisfaction of relatedness needs at school; Comp = satisfaction of competence needs at school; SS = school satisfaction. ^∗^*p* < 0.05, ^∗∗^*p* < 0.01, ^∗∗∗^*p* < 0.001.*

### The Measurement and Structural Models

We conducted a CFA to examine the validity of the measures used in the current study. The CFA results demonstrated that all measures had adequate fit.

#### The Model of Basic Psychological Needs at School as Direct Predictors of Prosocial Behavior and Antisocial Behavior

The measurement model consisted of five latent factors (T1 autonomy needs satisfaction at school, T1 relatedness needs satisfaction at school, T1 competence needs satisfaction at school, T3 prosocial behavior, T3 antisocial behavior), 28 observed variables, and two controlled variables (gender, age). A test of the measurement model showed an acceptable fit to the data: χ^2^(336) = 1094.04; CFI = 0.93; TLI = 0.92; SRMR = 0.04; RMSEA = 0.05 (90% CI for the RMSEA is 0.050–0.057). The measured variables’ loadings were all statistically significant (*p* < 0.001) on their latent variables, showing that the latent variables were adequately measured by their indicators.

**FIGURE 1:**
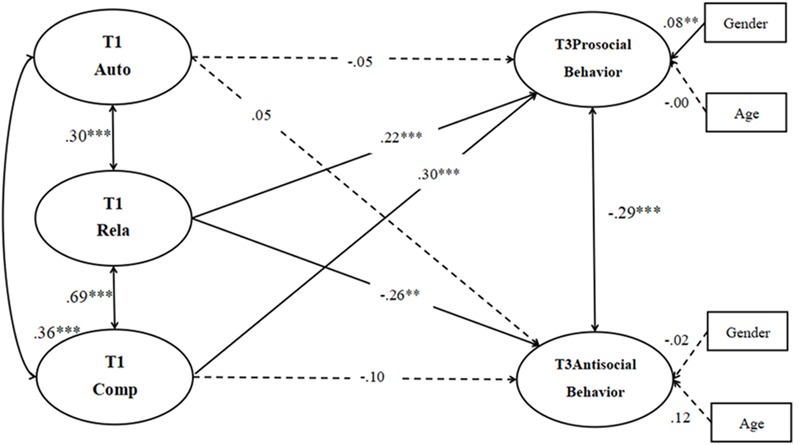
The model of basic psychological needs at school as direct predictors of prosocial and antisocial behavior (Model 1). Auto = satisfaction of autonomy needs at school; Rela *=* satisfaction of relatedness needs at school; Comp = satisfaction of competence needs at school; SS = school satisfaction. The numbers represent the path coefficients of the model; the significant paths are indicated with asterisks, the non-significant paths are represented by dotted lines. For simplicity, the observed variables are not presented in the figure. ^∗^*p* < 0.05, ^∗∗^*p* < 0.01, ^∗∗∗^*p* < 0.001.

A model considering all the paths revealed an acceptable fit to the data, χ^2^(388) = 1156.39; CFI = 0.93; TLI = 0.92; SRMR = 0.04; RMSEA = 0.05 (90% CI for the RMSEA is 0.046–0.053). Hypothesis 1 predicted that children’s satisfaction of basic psychological needs at school will directly predict their prosocial behavior and antisocial behavior. The coefficients of direct path in **Figure 1** showed that T1 satisfaction of relatedness needs at school and competence needs at school demonstrated a significantly positive effect on measures of T3 prosocial behavior respectively (β = 0.22, *p* < 0.001; β = 0.30, *p* < 0.001), with 21% of the variance explained for prosocial behavior. Only T1 satisfaction of relatedness needs at school demonstrated a significant and negative effect on T3 antisocial behavior (β = -0.26, *p* < 0.01), with 12% of the variance explained for antisocial behavior. T1 satisfaction of autonomy needs at school was unrelated to T3 prosocial behavior and T3 antisocial behavior. T1 satisfaction of competence needs at school was unrelated to T3 antisocial behavior, suggesting that satisfaction of competence needs at school may be a protective factor for prosocial behavior but not for antisocial behavior. Hence, Hypothesis 1 was partly supported. Furthermore, children’s gender revealed a significant effect on prosocial behavior, but not on antisocial behavior. However, children’s age levels showed no significant effect on prosocial or antisocial behavior.

#### The Mediation Model of Satisfaction of Basic Psychological Needs at School, School Satisfaction, Prosocial Behavior, and Antisocial Behavior

The measurement model consisted of six latent factors (T1 autonomy needs satisfaction at school, T1 relatedness needs satisfaction at school, T1 competence needs satisfaction at school, T3 prosocial behavior, T3 antisocial behavior, T2 school satisfaction), 34 observed variables and two controlled variables (gender, age). A test of the measurement model showed an acceptable fit to the data: χ^2^(506) = 1520.29; CFI = 0.92; TLI = 0.91; SRMR = 0.05; RMSEA = 0.05 (90% CI for the RMSEA is 0.047–0.053). The measured variables’ loadings were all statistically significant (*p* < 0.001) on their latent variables, showing that the latent variables were adequately measured by their indicators.

**FIGURE 2:**
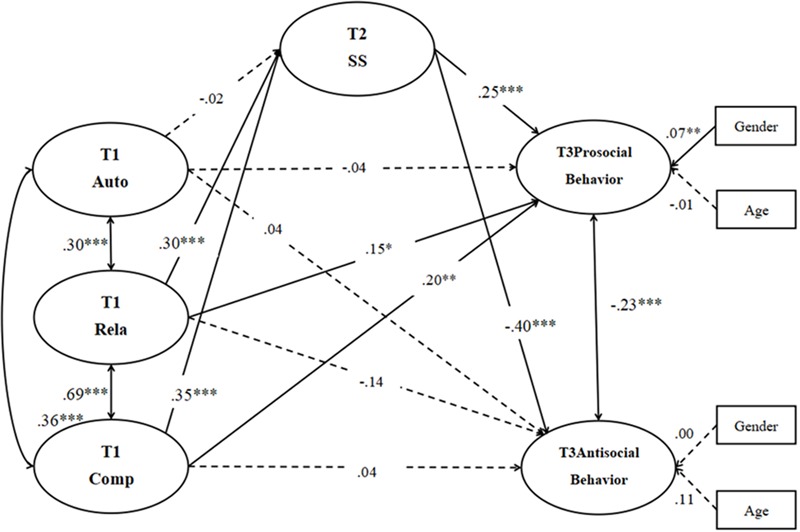
The simplified mediation model (Model 2). Auto = satisfaction of autonomy needs at school; Rela *=* satisfaction of relatedness needs at school; Comp = satisfaction of competence needs at school; SS = school satisfaction. The numbers represent the path coefficients of the model; the significant paths are indicated with asterisks, the non-significant paths are represented by dotted lines. For simplicity, the observed variables are not presented in the figure. ^∗^*p* < 0.05, ^∗∗^*p* < 0.01, ^∗∗∗^*p* < 0.001.

A model considering all the paths revealed an acceptable fit to the data, χ^2^(570) = 1596.03; CFI = 0.92; TLI = 0.91; SRMR = 0.05; RMSEA = 0.05 (90% CI for the RMSEA is 0.045–0.050). The path coefficients in **Figure 2** show that T2 school satisfaction was predicted by T1 satisfaction of relatedness needs at school and competence needs at school (β = 0.30, *p* < 0.001; β = 0.35, *p* < 0.001), but not T1 satisfaction of autonomy needs at school (β = -0.02, *p* > 0.05), with 35% of the variance explained for T2 school satisfaction. T1 satisfaction of relatedness needs at school and competence needs at school as well as T2 school satisfaction showed significantly positive effects on T3 prosocial behavior respectively (β = 0.15, *p* < 0.05; β = 0.20, *p* < 0.01; β = 0.25, *p* < 0.001), but not T1 satisfaction of autonomy needs at school (β = -0.04, *p* > 0.05), with a total of 25% of the variance explained for T3 prosocial behavior. Only T2 school satisfaction showed significantly negative effects on T3 antisocial behavior (β = -0.04, *p* < 0.001), but not T1 satisfaction of autonomy needs at school and relatedness needs at school as well as T1 satisfaction of competence needs at school (β = 0.04, *p* > 0.05; β = -0.14, *p* > 0.05; β = 0.04, *p* > 0.05), with a total of 29% of the variance explained for T3 antisocial behavior. Furthermore, children’s gender revealed significant effect on prosocial behavior, but not on antisocial behavior. However, children’s age levels showed no significant effect on prosocial or antisocial behavior.

### Mediating Effect of School Satisfaction

Hypothesis 2 predicted that children’s school satisfaction will mediate the relation between three basic psychological needs satisfaction at school and their prosocial behavior. The bootstrapping tests (Preacher and Hayes, 2008) confirmed that the 95% CI around the mediation effect of T2 school satisfaction in relation to T1 satisfaction of relatedness needs at school and competence needs at school (95% CI = [0.02, 0.13]; 95% CI = [0.03, 0.15]) did not contain zero, but the mediation effect of T2 school satisfaction in relation to T1 satisfaction of autonomy needs at school (95% CI = [-0.03, 0.01]) contained zero. These findings indicated that the mediation effect of T2 school satisfaction in relation to T1 satisfaction of relatedness needs at school and competence needs at school was significant, whereas the mediation effect of T2 school satisfaction in relation to T1 satisfaction of autonomy needs at school was not significant. The magnitudes of the mediating effect of T1 satisfaction of relatedness needs at school and competence needs at school on T3 prosocial behavior through T2 school satisfaction were 0.08 and 0.09 (*SE* = 0.03, *p* < 0.01; *SE* = 0.03, *p* < 0.01), respectively. Thus, Hypothesis 2 was partly supported.

Hypothesis 3 predicted that children’s school satisfaction will mediate the relation between the three basic psychological needs satisfaction at school and their antisocial behavior. The bootstrapping tests (Preacher and Hayes, 2008) confirmed that the 95% CI around the mediation effect of T2 school satisfaction in relation to T1 satisfaction of relatedness needs at school and competence needs at school (95% CI = [-0.20, -0.05]; 95% CI = [-0.26, -0.06]) did not contain zero, whereas the mediation effect of T2 school satisfaction in relation to T1 satisfaction of autonomy needs at school (95% CI = [-0.02, 0.04]) contained zero. These findings indicated that the mediation effect of T2 school satisfaction in relation to T1 satisfaction of relatedness needs at school and competence needs at school was significant, whereas the mediation effect of T2 school satisfaction in relation to T1 satisfaction of autonomy needs at school was not significant. The magnitudes of the mediating effects of T1 satisfaction of relatedness needs at school and competence needs at school on T3 antisocial behaviors through school satisfaction were -0.12 and -0.14 (*SE* = 0.04, *p* < 0.01; *SE* = 0.04, *p* < 0.01) respectively. Therefore, Hypothesis 3 was partly supported.

## Discussion

Using a three-wave longitudinal design, we investigated the differential direct and indirect predictive effects of the three basic psychological needs satisfaction at school and school satisfaction on prosocial and antisocial behavior among elementary school children. To a certain extent, our study thus provided a more comprehensive analysis of the directionality relations among those variables than previous studies that have been limited to cross-sectional and two-wave longitudinal studies. Consistent with the BPNT, our findings provided evidence supporting the perception that the satisfaction of basic psychological needs leads to more frequent positive social behavior in the school setting. Furthermore, the demonstration of the significant mediating role of school satisfaction illustrates the importance of a cognitive mechanism in the link between psychological needs satisfaction and social behavior in schools.

### Satisfaction of Basic Psychological Needs at School and Prosocial Behavior and Antisocial Behavior

The results revealed that among elementary school children, satisfaction of the three basic needs at school played different roles in the manifestation of prosocial and antisocial behavior. Specifically, the T1 satisfaction of relatedness needs at school and competence needs at school both displayed statistically significant direct effects on T3 prosocial behavior; however, the T1 satisfaction of autonomy needs at school displayed no significant direct effects on T3 prosocial behavior. The T1 satisfaction of relatedness needs at school also displayed direct effects on T3 antisocial behavior, while neither the T1 satisfaction of autonomy needs or competence needs at school displayed significant direct effects on T3 antisocial behavior.

We elaborated on the findings below. First, our findings suggest the importance of the satisfaction of relatedness and competence (but not autonomy) needs in facilitating children’s subsequent prosocial behavior in the school setting. These findings support the general tenets of the BPNT, which propose that the satisfaction of basic psychological needs leads to more adaptive behavioral outcomes and positive social functioning. Furthermore, the BPNT also theorizes that when a particular need is satisfied, additional engagement with experiences that satisfy that need will ensue (Ryan and Deci, 2000). Satisfaction of relatedness needs at school may thus be important in promoting children’s prosocial behavior because it increases their sense of closeness to teachers and peers (Kou and Wang, 2003; Weinstein and Ryan, 2010; Pavey et al., 2011). Also in line with the BPNT, satisfaction of competence needs at school may facilitate children’s later prosocial behavior because it reflects the extent to which children *expect* that they have the ability to do a favor for other people (e.g., teachers and peers in school), which fuels prosocial behavior demonstrating their *actual* ability to help someone (Caprara and Steca, 2005; Weinstein and Ryan, 2010). This finding is consistent with recent cross-sectional studies indicating that volunteers who are satisfying their competence needs show a higher degree of engagement in their volunteer work (Haivas et al., 2013; Jones et al., 2015).

The finding that differences in the satisfaction of autonomy needs did not predict later children’s prosocial behavior in school is consistent with previous research showing the relatively smaller contribution of autonomy needs relative to relatedness and competence needs in the prediction of prosocial behavior (Gagneì, 2003). One possible reason is that individuals might experience fluctuation in the satisfaction of the three needs, while different situation of need satisfaction would have different effects on the outcome variables. Recent studies showed a significant increase in the satisfaction of autonomy needs from Grades 6 to 9 (Taylor et al., 2010). Furthermore, a longitudinal study revealed that adolescents in trajectories characterized by high psychological needs satisfaction evidenced the highest levels of social, academic, and personal–emotional adjustment at the end of high school (Ratelle and Duchesne, 2014). In addition, researchers have pointed out that a non-significant correlation between the needs for autonomy and prosocial behavior with a negative parameter estimate approaching significance may indicate suppression effects (Maassen and Bakker, 2001; Morgan, 2016), which means that although the satisfaction of autonomy needs at school did not directly influence children’s prosocial behavior, it still could influence prosocial behavior through potential mediators (MacKinnon, 2008; Quoidbach et al., 2010; Wen and Ye, 2014; Morgan, 2016). This possibility may offer another explanation for our findings.

Second, our findings revealed that the satisfaction of relatedness needs, but not competence or autonomy needs at school, significantly predicted antisocial behavior in the longitudinal study, indicating that children whose relatedness needs remain relatively unsatisfied would display more antisocial behavior in school. Our finding is supported by a longitudinal study, which showed that poor relationships with peers and teachers led to more serious forms of antisocial behavior in elementary school (Snyder et al., 2005).

Our findings regarding the needs for autonomy at school and competence at school may indicate that differences in the satisfaction of these needs may indeed *not* relate to later antisocial behavior. However, the findings may also reflect the suppression effects. For example, researchers have revealed that antisocial tendencies may emerge in children experiencing low autonomy support and maltreatment through disruptions to a child’s behavioral control systems (Thibodeau et al., 2015). Moreover, our finding about the lack of a relation between the needs for competence at school and antisocial behavior by a longitudinal design is supported by a cross-sectional study showing that athletes’ satisfaction of competence needs had an inverse relation with antisocial behavior, although there was no significant direct influence on antisocial behavior (Hodge and Gucciardi, 2015). Such findings suggest that suppression effects may be involved in the thwarting of competence needs at school and antisocial behavior. Theoretically, the BPNT hypothesizes that if individuals are unable to obtain basic need satisfaction, they are likely to engage in activities that satisfy less authentic, substitute needs, which may operate as mediators as well (Deci and Ryan, 2011). Based on the BPNT, further research may be beneficial to clarify the possible complex relations among these variables.

### The Mediation Effect of School Satisfaction

The hypothesized mediation effects of school satisfaction in the association between satisfaction of basic psychological needs at school and prosocial and antisocial behavior were partially supported in our longitudinal study. Specifically, both the T1 satisfaction of relatedness needs at school and competence needs at school displayed indirect effects on T3 prosocial and antisocial behavior via T2 school satisfaction whereas the T1 satisfaction of autonomy needs at school failed to have indirect effects on T3 prosocial or antisocial behavior via T2 school satisfaction.

First, that school satisfaction mediated the relation between satisfaction of relatedness need at school and competence needs at school and prosocial behavior among children suggested that children who feel connected to their teachers and peers and feel competent in their capabilities in school tend to be more content with their school lives, and in turn express more prosocial behavior later. Our findings are in line with the self-system process model, which theorizes that individuals actively appraise needs satisfaction across environments, and when needs are satisfied within particular contexts such as school, engagement occurs in affect, behavior, and cognition (Connell and Wellborn, 1991). Our findings are also consistent with studies showing that children whose relatedness needs and competence needs are satisfied tended to report positive perceptions of school (Cock and Halvari, 1999; Osterman, 2000; Verkuyten and Thijs, 2002). Thus, our study extended the existing literature of prosocial behavior and satisfaction by identifying school satisfaction as a key cognitive mediator in the relations between the satisfaction of relatedness and competence needs and children’s prosocial behavior in school.

Second, our study also supported the expectation that school satisfaction would mediate the relations between relatedness needs satisfaction at school and competence needs satisfaction at school, and elementary school children’s antisocial behavior. In agreement with previous research showing that positive youth development enabled students’ positively affective and cognitive evaluations of school context and then reduced their misbehavior (Sun, 2016), our study found that school satisfaction was a potential protective psychological strength that could be increased by the satisfaction of basic psychological needs at school, subsequently alleviating children’s antisocial behavior, similar to the function of life satisfaction (Suldo and Huebner, 2004). Our results also extended the existing literature of antisocial behavior and satisfaction by demonstrating that school satisfaction played a stronger role in preventing children’s antisocial behavior in school through influencing the relations among satisfaction of relatedness needs at school and competence needs at school, and antisocial behavior. In total, our results reveal an important mediation mechanism between need satisfaction and later social behavior in school by using longitudinal research design, supporting the critical role of school satisfaction in influencing children’s adaptive social behavior in school. We also found differences in the predictors of prosocial and antisocial behavior, showing more important roles for the needs for relatedness and competence in the development of prosocial behavior along with the more important role of the need for relatedness in the development of antisocial behavior.

However, we found that school satisfaction failed to mediate the relation between satisfaction of autonomy needs at school and prosocial or antisocial behavior. Our results revealed that the satisfaction of autonomy needs may, directly or indirectly, play a relatively less important role in influencing social behavior among elementary school-age children. In a previous study, life satisfaction was also found to be unrelated to prosocial behavior (Anderson, 2013), while the need for autonomy was also discovered to be unrelated to prosocial behavior (Morgan, 2016), which partially supports our findings. In addition, it is possible that autonomy needs satisfaction at school, in concert with the other two basic psychological needs satisfaction at school, is required to promote prosocial action and reduce antisocial behavior. Although the constructs are distinguishable, there is overlap among them (Boezeman and Ellemers, 2009; Haivas et al., 2013). Although in theory it may be possible for one need to be satisfied while one or the other two needs is not, in reality it may be likely that the satisfaction of one need arises in conjunction with the satisfaction of one or the other two needs. Such a possibility may offer another explanation for our findings. Further research that examines methods of activating the three needs in unison could complement our study.

### Limitations and Future Research

Our study had limitations that should be acknowledged. First, our study did not control for baseline levels of prosocial or antisocial behavior, limiting the determination of causal relationships among the variables. Multi-wave studies that assess key variables at each wave are thus needed. Second, our study was limited to the use of self-report measures. Further research should complement multiple sources of data to minimize common method bias, for example, collecting the reports of students’ important informants, such as teachers and peers. Third, we also found significant gender effect on prosocial behavior, but not on antisocial behavior in two models. The gender effect shows that boys were expected to exhibit higher levels of prosocial behavior than girls, which offers more evidence regarding the equivocal findings on gender differences in the past (Eisenberg and Fabes, 1998; de Guzman et al., 2005). Because children might underreport sensitive personal information, such as the frequency and severity of one’s antisocial behavior, the effects of social desirability should be taken into consideration. Fourth, we conducted our study with Chinese children. Therefore, our results may not extend to students from other cultural backgrounds. Future research should test the model among students from other cultures, such as more individualistic cultures.

### Implications

Our study has important practical implications for school professionals in elementary school settings. Despite the youthfulness of elementary school children, it is not too early for school professionals to incorporate into the curriculum empirically-based assessment methods and prevention and intervention programs that focus on social behavior. Given that both needs for relatedness and competence related to school satisfaction and one or more aspects of social behavior, the most effective efforts likely will address both social behavior and school satisfaction in school. Examples of such programs (e.g., Positive Behaviors Supports) can be found in previous studies (Lewis et al., 2002; Hawken, 2014). First, similarly, programs involving positive psychological needs supports could be implemented in schools to provide sufficient opportunities for satisfying children’s needs for relatedness and competence at school (Zhang et al., 2011). Then, schools could identify clear and brief behavioral expectations, encourage the appropriate behaviors, and provide appropriate supports. Finally, creating an inviting, developmentally appropriate school environment is essential for enhancing students’ satisfaction with school (Baker et al., 2003). Thus, educators and psychologists can gain some insight from this study for improving young children’s school environments through the identification of key antecedents of their social behavior, including the presence of prosocial behavior and the absence of antisocial behavior.

## Author Contributions

LT: analyzed and interpreted the data; prepared the draft and the contributing authors (XZ and EH) reviewed it critically and gave important intellectual content. LT and XZ: participated in the acquisition of data. All the authors worked for the final approval of the version to be published; accountable for all the aspects of the work in ensuring that questions related to the accuracy or integrity of any part of the work are appropriately investigated and resolved; substantially contributed to the conception and the design of the work.

## Conflict of Interest Statement

The authors declare that the research was conducted in the absence of any commercial or financial relationships that could be construed as a potential conflict of interest.
